# Efficacy and safety of ashwagandha root extract in children and adolescents with mild attention-deficit/hyperactivity disorder: a randomized, double-blind, placebo-controlled trial

**DOI:** 10.25122/jml-2026-0025

**Published:** 2026-06

**Authors:** Sunil Naik, Gudla Muralidhar, Bade Srinivas Rao, Sanjay Agarwal, Mukta Jain

**Affiliations:** 1 Government Medical College and Government General Hospital, Srikakulam, Andhra Pradesh, India; 2 Department of Community Medicine, D.Y Patil University, School of Medicine, Nerul-Navi Mumbai, Maharshtra, India; 3 Mahatma Gandhi Mission Medical College, Nerul, Navi Mumbai, India

**Keywords:** ashwagandha root extract, attention deficit hyperactivity disorder, BRIEF-2, PROMIS, CGI, children, ADHD, attention-deficit/hyperactivity disorder, ADHD-RS-IV, Attention-Deficit/Hyperactivity Disorder Rating Scale-IV, ARE, ashwagandha root extract, BRIEF-2, Behaviour Rating Inventory of Executive Function-2, BRI, Behavioural Regulation Index, CGI, Clinical Global Impression, CRI, Cognitive Regulation Index, CONSORT, Consolidated Standards of Reporting Trials, CTRI, Clinical Trials Registry of India, DSM-5, Diagnostic and Statistical Manual of Mental Disorders, Fifth Edition, GEC, Global Executive Composite, HPA, hypothalamic-pituitary-adrenal, HPLC, high-performance liquid chromatography, IA, inattention, ICH-GCP, International Council for Harmonisation Good Clinical Practice, ITT, intention-to-treat, HI, hyperactivity-impulsivity, PL, placebo, PP, per-protocol, PROMIS, Patient-Reported Outcomes Measurement Information System

## Abstract

This study investigated the effects of ashwagandha root extract (ARE) supplementation on core attention-deficit/hyperactivity disorder (ADHD) symptoms, executive functioning, and patient-reported outcomes in children and adolescents with mild ADHD. Fifty-eight children and adolescents with clinically diagnosed mild ADHD were randomly assigned to either the ARE group (ARE; *n* = 29) or a placebo group (PLA; *n* = 29) in a prospective, randomized, double-blind, placebo-controlled, parallel-group design. Participants received 150 mg of ashwagandha root extract or a matched placebo gummies twice daily for 56 days. Primary outcomes were changes in the Attention-Deficit/Hyperactivity Disorder Rating Scale-IV (ADHD-RS-IV) total score. Secondary outcomes: ADHD-RS-IV inattention and hyperactivity–impulsivity subscales, Behavior Rating Inventory of Executive Function-2 (BRIEF-2) scores and Patient-Reported Outcomes Measurement Information System (PROMIS) scores. Safety markers, including routine clinical laboratory parameters, were assessed at baseline and Day 56. At Day 56, ADHD-RS-IV inattention, hyperactivity-impulsivity, and total scores were significantly lower in ARE than in PLA (*P* < 0.001). Significant improvements were also observed in the BRIEF-2 behavioral, emotional, and cognitive regulation indices and the global executive composite in ARE (*P* < 0.001). PROMIS sleep disturbance and anxiety scores improved significantly in ARE relative to PLA (*P* < 0.001). No significant between-group differences or clinically meaningful changes were observed in safety markers across the intervention period. No serious adverse events were reported. Supplementation with 300 mg/day of ashwagandha root extract for 56 days significantly improved ADHD symptom severity, executive functioning, and selected patient-reported outcomes in children and adolescents with mild ADHD, with a favorable safety profile.

## Introduction

Attention-Deficit/Hyperactivity Disorder (ADHD) is a neurodevelopmental disorder in childhood, characterized by persistent inattention, hyperactivity, and impulsivity [1] that interfere with educational, social, and family settings [2]. Global prevalence estimates suggest that approximately 7-8% of children aged 3-12 years and 5-6% of adolescents meet diagnostic criteria according to the Diagnostic and Statistical Manual of Mental Disorders, Fifth Edition (DSM-5) [3]. Symptoms typically emerge in the early school years and may persist, in full or in part, into adolescence and adulthood [4]. Core behavioral manifestations are closely associated with executive dysfunction, including deficits in working memory, sustained attention, and inhibitory control, contributing to poorer academic achievement and broader functional difficulties [5].

Current ADHD management guidelines recommend multimodal treatment strategies, combining pharmacological therapy with psychosocial interventions such as parent training and school-based behavioral support [6]. Stimulant medications (e.g., methylphenidate, amphetamine formulations) and non-stimulant agents (e.g., atomoxetine, alpha-agonists) demonstrate short- to medium-term efficacy in reducing core symptoms [7]. However, long-term safety and sustained effectiveness remain incompletely understood. Psychosocial approaches are often implemented adjunctively or when medication is contraindicated [8]. Despite these options, treatment challenges persist, including suboptimal response, adverse effects, residual impairment, and limited evidence supporting prolonged benefit [9].

Recognition of these limitations has prompted growing interest in complementary and integrative strategies [10]. Herbal interventions targeting cognitive performance, stress regulation, and neuropsychological well-being have received increasing attention [11]. Adaptogenic botanicals such as ashwagandha root extract have been investigated primarily in adults for anxiolytic, neuroprotective, and cognitive-enhancing effects [12]. Traditionally used in Ayurvedic medicine to promote physiological and psychological resilience [13], ashwagandha’s mechanisms are thought to involve modulation of stress-related pathways, including the hypothalamic–pituitary–adrenal (HPA) axis and neurotransmitter systems relevant to cognition and emotional regulation [14].

Clinical studies in adults report reductions in perceived stress and cortisol levels, alongside improvements in sleep quality, physical performance, wellbeing, and selected cognitive domains following supplementation with standardized extracts [15-18]. However, most research has involved healthy adults or individuals with chronic stress rather than pediatric or neurodevelopmental populations. Evidence in children and adolescents, particularly those diagnosed with ADHD, remains limited. Few controlled trials have examined ashwagandha as a standalone intervention in pediatric populations; one study reported improvements in children with malnutrition [19]. Existing investigations often assess general psychological well-being rather than core ADHD domains such as sustained attention, impulsivity, and hyperactivity [20].

A recent systematic review identified twenty-nine clinical trials evaluating herbal products for mental health outcomes in children and adolescents, including ADHD, anxiety, and mood disorders [21]. Findings suggested preliminary safety and potential efficacy, supported by bioinformatic analyses indicating multitarget mechanisms involving neuroprotection, modulation of neuroinflammation, and neurotransmission. Nevertheless, the authors emphasized the need for standardized extracts, rigorous placebo-controlled designs, clarification of optimal dosing, and long-term safety evaluation [21]. Accordingly, high-quality trials assessing ashwagandha root extract monotherapy in pediatric ADHD are lacking.

Ashwagandha contains diverse bioactive constituents, including alkaloids, withanolides, sitoindosides, and steroidal lactones [22]. Root-derived extracts are considered preferable, as potentially cytotoxic compounds, such as *withaferin A* and *withanone*, are more concentrated in leaves [23-28]. Although these compounds exhibit anticarcinogenic activity in experimental models, excessive exposure has been associated with hepatotoxicity [28].

The present study, therefore, evaluates a standardized, root-only ashwagandha extract (KSM-66; Ixoreal Biomed Inc., Los Angeles, California, USA), produced using a green, aqueous-based extraction process and standardized to approximately 5% withanolides by high-performance liquid chromatography (HPLC). Administered at 150 mg twice daily, the study aims to determine its effects on core ADHD symptoms, cognitive performance, and physiological stress markers compared with placebo.

## Material and Methods

### Study design

The study followed a prospective, randomized, double-blind, placebo-controlled, parallel-group design over 56 days. Participants were children and adolescents diagnosed with ADHD and were enrolled at a single clinical center. Eligible participants were randomly assigned to one of two study arms to receive either ashwagandha gummies (150 mg) or a matched placebo, allowing for a comparative assessment of efficacy and safety.

### Participants

Children and adolescents with a clinical diagnosis of ADHD were recruited from the outpatient clinic at the study site. Potential participants were screened for eligibility based on predefined inclusion and exclusion criteria, medical history, and safety considerations. Written informed consent was obtained from parents or legal guardians before any study procedures, and assent was obtained from all participating children and adolescents in accordance with ethical requirements.

**Figure 1:**
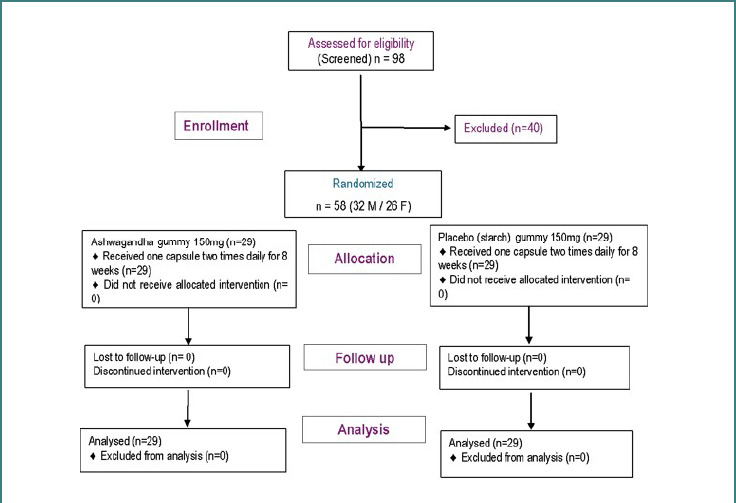
CONSORT flow representation of the patient enrolment, allocation, follow-up, and analysis

Fifty-eight participants were randomized to ashwagandha root extract (ARE; *n* = 29) or placebo (*n* = 29). Children aged 6–12 years of both sexes and all ADHD subtypes were included. Baseline demographics, socioeconomic, and clinical characteristics were comparable between groups, with no significant differences. The Consolidated Standards of Reporting Trials (CONSORT) flow diagram is presented in Figure 1.

Participants were randomized 1:1 to ashwagandha root extract or placebo using a computer-generated schedule prepared by an independent statistician. Allocation numbers were assigned sequentially after eligibility confirmation. The trial was double-blind, with investigators and staff unaware of treatment codes until database lock. Emergency unblinding was allowed only for medical necessity and was documented. Three analysis sets were defined: safety (≥1 dose), intention-to-treat (≥1 dose plus post-baseline primary assessment), and per-protocol (no major deviations). Primary efficacy was analyzed in the intention-to-treat (ITT) population, and safety in the safety population.

Children and adolescents were eligible for inclusion with mild ADHD diagnosed according to DSM-IV criteria, confirmed by clinician assessment and structured parent interview, were eligible for inclusion. Participants were required to be free from stimulant or non-stimulant ADHD medications throughout the study. Exclusion criteria included comorbid psychiatric disorders, recent vitamin or nutritional supplement use, and participation in another clinical trial.

### Procedures

Eligible participants entered a 56-day study period consisting of two on-site visits and one interim telephone follow-up. All procedures were conducted under the investigator’s supervision and in accordance with the approved study protocol.

At baseline (Day 1), written parental consent and child assent were obtained before procedures. Demographics, medical and surgical history, and medication use were recorded. Physical examination and vital signs were assessed. Blood samples were collected for routine hematological and biochemical laboratory evaluations.

Baseline efficacy assessments included the validated ADHD Rating Scale-IV [29], administered by the investigator via structured interviews with parents, and the Behavior Rating Inventory of Executive Function-2 (BRIEF-2) parent version, which has demonstrated validity and reliability in clinical settings [30]. The valid and reliable Patient-Reported Outcomes Measurement Information System (PROMIS) questionnaires, assessed by parents, assessed sleep, anxiety, and quality of life and were completed at baseline [31]. Following completion of all baseline assessments, eligible participants were randomized 1:1 to receive either ashwagandha root extract gummies (150 mg) or identical placebo gummies.

Participants and caregivers were instructed to administer 1 gummy orally twice daily, after breakfast and dinner, with water for 56 consecutive days. Participants were advised to continue their usual diet and physical activity throughout the study period, unless otherwise directed by the investigator. Compliance with the dosing regimen was monitored through caregiver reporting and assessment of returned study supplementation.

An interim telephone follow-up was conducted on Day 28 to assess protocol adherence, treatment compliance, concomitant medication use, and any adverse events. At the end-of-study visit (Day 56), participants returned to the study site for a repeat physical examination, assessment of vital signs, and routine clinical laboratory testing. Efficacy assessments of the ADHD Rating Scale-IV, BRIEF-2 parent version, and PROMIS questionnaires were repeated. Overall clinical change and treatment response were evaluated using the Clinical Global Impression (CGI) scale [32], completed by the investigator. Adverse events and concomitant medication use were reviewed and documented throughout the study period until completion. Treatment adherence was confirmed through review of dosing records and returned study medication.

### Ethical committee

The study was conducted in accordance with the International Council for Harmonization Good Clinical Practice (ICH-GCP) (E6 R2, Step 4, 2016), the Declaration of Helsinki (Taipei 2016), and the regulatory framework outlined in the New Drugs and Clinical Trials Rules 2019 (India). The study-related documents were approved by the Institutional Ethics Committee (ECR/492/Inst/AP/2013/RR-20). The study was registered with the Clinical Trials Registry of India (CTRI #: CTRI/2022/02/040555; dt.23/02/2022). The study adhered to the Consolidated Standards of Reporting Trials (CONSORT) guidelines.

Written informed consent was obtained from the parents or legal guardians of all participating children and adolescents before any study-related procedures were performed. Age-appropriate assent was also obtained from participants where applicable. Investigators provided a full explanation of the study procedures, potential risks, and benefits to participants and their guardians before enrolment.

Serious adverse events included death, life-threatening events, hospitalization, disability, or congenital anomalies. Unexpected events were inconsistent with product information. Significant events included major laboratory abnormalities or those requiring intervention. Participants discontinuing due to adverse events were followed until resolution. Serious adverse events were reported within 24 hours.

### Statistical analysis

Sample size estimation was conducted using G*Power (version 3.1.9.7), with ADHD-RS-IV total score as the primary outcome. Assuming a between-group difference of 11.2 points in change from baseline and a common standard deviation of 14.0, 48 participants (24 per group) were required to achieve 85% power with a two-sided t-test at α = 0.05. To account for potential attrition, the target sample was increased to 58. The anticipated treatment effect corresponded to an effect size of 0.80, consistent with prior pediatric ADHD trials.

The primary endpoint was the change from baseline (last pre-dose assessment at Visit 1, Day 1) to Week 8 (Visit 3) in ADHD-RS-IV total score. The null hypothesis assumed no between-group difference in mean change at Week 8; the alternative hypothesis assumed a difference between ARE and placebo.

Descriptive statistics summarised baseline and outcome variables (Table 1). Categorical variables were analyzed using chi-square tests, and continuous outcomes with parametric methods. Missing data were not imputed. Participant disposition was summarised by treatment group, including screening, randomization, completion, and discontinuation.

**Table 1:** Baseline demographic profile

		ARE (*n* = 29)		Placebo (*n* = 29)		Chi-square test	
	*n*	No.	%	No.	%	χ^2^	*P*
**Gender**							
Male	32	15	51.7%	17	58.6%	0.279	0.597
Female	26	14	48.3%	12	41.4%		
**Age groups**							
6 yrs.	3	2	6.9%	1	3.4%	6.035	0.419
7 yrs.	7	3	10.3%	4	13.8%		
8 yrs.	7	2	6.9%	5	17.2%		
9 yrs.	12	4	13.8%	8	27.6%		
10 yrs.	11	8	27.6%	3	10.3%		
11 yrs.	6	4	13.8%	2	6.9%		
12 yrs.	12	6	20.7%	6	20.7%		
**Parents education**							
Bachelor’s degree	29	12	41.4%	17	58.6%	1.788	0.409
Up to Secondary School	2	1	3.4%	1	3.4%		
Higher secondary School	27	16	55.2%	11	37.9%		
**Annual family income**							
5 to 15 (INR Lakh)	14	4	13.8%	10	34.5%	3.390	0.066
<5 (INR Lakh)	44	25	86.2%	19	65.5%		
**Type of ADHD**							
Combined	33	14	48.3%	19	65.5%	2.073	0.355
Hyperactive/Impulsive	19	12	41.4%	7	24.1%		
Inattentive	6	3	10.3%	3	10.3%		
**Medical history**							
Anxiety	18	8	27.6%	10	34.5%	4.038	0.544
Depression	11	8	27.6%	3	10.3%		
Hypersomnia	8	4	13.8%	4	13.8%		
Hyposomnia	4	1	3.4%	3	10.3%		
Insomnia	10	4	13.8%	6	20.7%		
Loss of Energy	7	4	13.8%	3	10.3%		

* ADHD, Attention Deficit/Hyperactivity Disorder; ARE, ashwagandha root extract; INR, Indian Rupees; Lakh, 100,000 Indian Rupees; Hypersomnia, excessive sleep duration or increased daytime sleepiness; Hyposomnia, sleep duration below age-appropriate normative ranges. Significance values were calculated using chi-square tests to assess differences between groups.

## Results

### ADHD Rating Scale-IV

**Figure 2:**
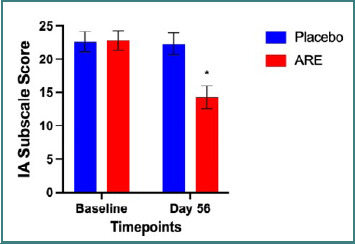
ADHD-RS-IV inattention score ARE, Ashwagandha root extract; IA, Inattention

**Figure 3:**
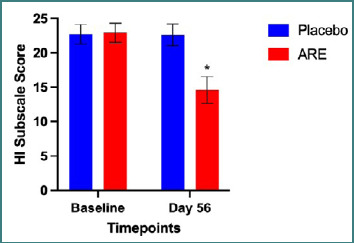
ADHD-RS-IV Hyperactivity-Impulsivity Score ARE, Ashwagandha root extract; HI, Hyperactivity-impulsivity

**Figure 4:**
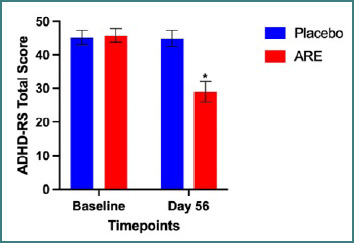
ADHD-RS-IV total score ARE, Ashwagandha root extract; ADHD-RS-IV, Attention-Deficit/Hyperactivity Disorder Rating Scale Version IVn

Significance was set at *P* < 0.05 (mean ± SD; Table 2). Baseline ADHD-RS-IV Inattention (IA), Hyperactivity-Impulsivity (HI), and total scores were comparable between groups (all *P* > 0.05). At Day 56, IA scores were significantly lower with ARE versus placebo (14.3 ± 1.7 vs 22.3 ± 1.6; p < 0.001; Figure 2), with a significant reduction only in ARE (*P* < 0.001) and no change in placebo (*P* = 0.243). HI scores were also reduced with ARE (14.6 ± 1.9 vs 22.6 ± 1.6; *P* < 0.001; Figure 3), improving only in ARE (*P* < 0.001; placebo *P* = 0.703). Total scores were lower with ARE (29.0 ± 3.0 vs 44.9 ± 2.5; *P* < 0.001; Figure 4), with significant improvement in ARE and no change in placebo (*P* = 0.217).

**Table 2:** ADHD Rating Scale-IV and PROMIS Scores in two groups

	ARE (*n* = 29)	Placebo (*n* = 29)	P* (between group)	Effect size
	Mean ± SD	Mean ± SD		Cohen’s ‘d’
**IA subscale score**				
Baseline	22.8 ± 1.5	22.6 ± 1.5	0.478	
Day 56	14.3 ± 1.7	22.3 ± 1.6	<0.001	
Change from baseline on day 56	-8.5 ± 2.1	-0.2 ± 1.1	<0.001	3.952
P** (within treatment)	<0.001	0.243		
**HI subscale score**				
Baseline	22.9 ± 1.4	22.7 ± 1.4	0.519	
Day 56	14.6 ± 1.9	22.6 ± 1.6	<0.001	
Change from baseline on day 56	-8.3 ± 2.3	-0.1 ± 1.4	<0.001	3.565
P** (within treatment)	<0.001	0.703		
**ADHD-RS total score**				
Baseline	45.8 ± 2.1	45.2 ± 2.2	0.367	
Day 56	29.0 ± 3.0	44.9 ± 2.5	<0.001	
Change from baseline on day 56	-16.8 ± 0.7	-0.3 ± 0.3	<0.001	23.571
P** (within treatment)	<0.001	0.217		
**PROMIS scores in two groups**				
**Sleep disturbance score**				
Baseline	32.6 ± 2.6	32.3 ± 2.5	0.719	
Day 56	19.7 ± 2.4	33.0 ± 2.1	<0.001	
Change from baseline on day 56	-12.9 ± 3.3	0.7 ± 1.4	<0.001	4.121
P** (within treatment)	<0.001	0.011		
**Anxiety score**				
Baseline	32.7 ± 2.6	32.2 ± 2.4	0.531	
Day 56	19.3 ± 3.2	33.1 ± 2.1	<0.001	
Change from baseline on day 56	-13.3 ± 3.8	0.8 ± 1.3	<0.001	3.710
P** (within treatment)	<0.001	0.002		
**Total score**				
Baseline	65.2 ± 3.4	64.6 ± 5.0	0.447	
Day 56	39.0 ± 4.5	66.1 ± 2.8	<0.001	
Change from baseline on day 56	-26.2 ± 1.0	1.5 ± 0.3	<0.001	27.700
P** (within treatment)	<0.001	<0.001		

* Independent sample *t*-test; **Paired sample t-test; ADHD-RS-IV, Attention-Deficit/Hyperactivity Disorder Rating Scale Version IV; ARE, ashwagandha root extract; PROMIS, Patient-Reported Outcomes Measurement Information System; HI, Hyperactivity-impulsivity; IA, Inattention; SD, Standard Deviation

### BRIEF-2

**Figure 5:**
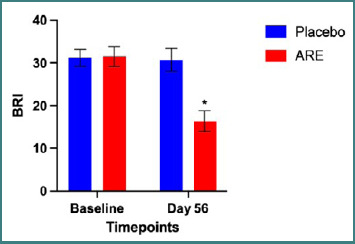
BRIEF-2 Behavioural Regulation Index Score ARE, Ashwagandha root extract; BRI, Behavioural Regulation Index

**Figure 6:**
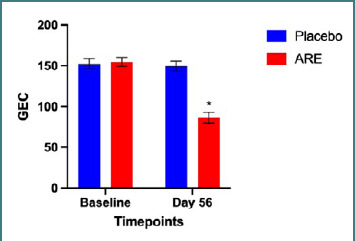
BRIEF-2 Global Executive Composite Score ARE, Ashwagandha root extract; GEC, Global Executive Composite

Baseline BRIEF-2 index scores were comparable between groups for the Behavioral Regulation Index (BRI), Emotional Regulation Index (ERI), Cognitive Regulation Index (CRI), and Global Executive Composite (GEC) (all *P* > 0.05) (Table 3). At Day 56, BRI scores were significantly lower in the ARE group than in the placebo group (16.4 ± 2.4 vs 30.7 ± 2.7; *P* < 0.001; Figure 5), with a significant reduction in ARE (*P* < 0.001) and no change in placebo (*P* = 0.418). ERI scores were also lower with ARE (25.5 ± 4.0 vs 39.5 ± 2.9; *P* < 0.001), improving with ARE (*P* < 0.001) and not changing with placebo (*P* = 0.057). CRI (44.2 ± 3.6 vs 79.2 ± 3.3; *P* < 0.001) and GEC scores (86.1 ± 6.7 vs 149.4 ± 6.1; *P* < 0.001; Figure 6) showed similar significant reductions in ARE, with no change in placebo (all *P* > 0.05).

**Table 3:** BRIEF-2 scores in two groups

	ARE (*n* = 29)	Placebo (*n* = 29)	P* (between group)	Effect size
	Mean ± SD	Mean ± SD		Cohen’s ‘d’
**BRI**				
Baseline	31.5 ± 2.3	31.2 ± 2.0	0.626	
Day 56	16.4 ± 2.4	30.7 ± 2.7	<0.001	
Change from baseline on day 56	-15.1 ± 2.6	-0.5 ± 3.6	<0.001	4.055
P** (within treatment)	<0.001	0.418		
**ERI**				
Baseline	41.6 ± 2.9	41.1 ± 2.8	0.522	
Day 56	25.5 ± 4.0	39.5 ± 2.9	<0.001	
Change from baseline on day 56	-16.1 ± 0.8	-1.6 ± 0.8	<0.001	18.125
P** (within treatment)	<0.001	0.057		
**CRI**				
Baseline	81.4 ± 4.5	79.9 ± 4.9	0.237	
Day 56	44.2 ± 3.6	79.2 ± 3.3	<0.001	
Change from baseline on day 56	-31.2 ± 0.7	-0.7 ± 1.2	<0.001	24.583
P** (within treatment)	<0.001	0.558		
**GEC**				
Baseline	154.4 ± 5.4	152.2 ± 6.6	0.162	
Day 56	86.1 ± 6.7	149.4 ± 6.1	<0.001	
Change from baseline on day 56	-68.3 ± 6.7	-2.8 ± 10.0	<0.001	6.650
P** (within treatment)	<0.001	0.139		

* Independent sample *t*-test; **Paired sample t-test; BRI, Behavioural Regulation Index; BRIEF-2, Behavioural Regulation Index Executive Function - (Version 2.0); CRI, Cognitive Regulation Index; ERI, Emotional Regulation Index; GEC, Global Executive Composite; PROMIS, Patient-Reported Outcomes Measurement Information System; SD, Standard deviation

### PROMIS

**Figure 7:**
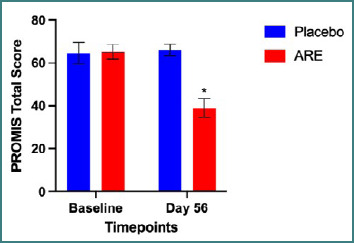
PROMIS total score ARE, Ashwagandha root extract; PROMIS, Patient-Reported Outcomes Measurement Information System

Baseline PROMIS sleep disturbance, anxiety, and total scores were comparable between groups (all *P* > 0.05) (Table 2). At Day 56, sleep disturbance scores were significantly lower with ARE versus placebo (19.7 ± 2.4 vs 33.0 ± 2.1; *P* < 0.001), with significant within-group reduction in ARE (*P* < 0.001) and a smaller but significant within-group decrease in placebo (*P* = 0.011). Anxiety scores were also lower with ARE (19.3 ± 3.2 vs 33.1 ± 2.1; *P* < 0.001), indicating a significant within-group reduction with ARE (*P* < 0.001) but a significant within-group increase with placebo (*P* = 0.002). PROMIS total scores were lower with ARE (39.0 ± 4.5 vs 66.1 ± 2.8; *P* < 0.001; Figure 7), with significant within-group changes in both groups (*P* < 0.001).

### Clinical laboratory parameters

No significant within-group changes from baseline to Day 56 were observed in the ARE or placebo groups for hematological or biochemical parameters (all *P* > 0.05; Table 4). Between-group comparisons at Day 56 were also non-significant. No clinically meaningful laboratory changes occurred in either group.

**Table 4:** Clinical laboratory parameters at baseline and day 56 in two groups

	ARE (*n* = 29)			Placebo (*n* = 29)			Between group
	Baseline	Day 56	Paired	Baseline	Day 56	Paired	
	Mean ± SD	Mean ± SD	P*	Mean ± SD	Mean ± SD	P*	P**
Hemoglobin (g/Dl)	13.0 ± 1.6	13.1 ± 1.5	0.850	13.0 ± 1.2	13.0 ± 1.1	0.945	0.734
RBC count (mil/cmm)	4.5 ± 0.4	4.5 ± 0.3	0.999	4.6 ± 0.4	4.7 ± 0.4	0.691	0.099
PCV (%)	39.2 ± 4.1	39.3 ± 4.0	0.997	38.7 ± 3.5	38.7 ± 3.5	0.982	0.591
TLC (cells/cmm)	6402.2 ± 1517.4	6629.4 ± 1737.0	0.598	6823.0 ± 1475.0	6790.0 ± 1334.8	0.929	0.695
Lymphocytes (%)	43.0 ± 4.2	43.0 ± 4.0	0.999	42.4 ± 4.5	42.2 ± 4.3	0.890	0.458
Monocytes (%)	6.7 ± 2.0	6.6 ± 2.0	0.891	6.1 ± 1.9	5.8 ± 1.7	0.431	0.092
Eosinophils (%)	2.6 ± 0.8	2.7 ± 0.4	0.418	2.3 ± 0.8	2.4 ± 0.7	0.924	0.018
Basophils (%)	0.6 ± 0.4	0.6 ± 0.4	0.741	0.3 ± 0.4	0.5 ± 0.5	0.123	0.339
ANC (cells/cmm)	3003.1 ± 766.5	3108. 2 ± 865.9	0.626	3296.0 ± 639.1	3324. 6 ± 639.3	0.866	0.284
Neutrophil %	47.1 ± 5.6	47.0 ± 5.5	0.980	48.8 ± 4.9	49.1 ± 4.7	0.799	-
Platelet Count (lakh/cmm)	3.4 ± 1.1	3.3 ± 1.2	0.873	3.2 ± 1.0	3.2 ± 1.0	0.794	0.515
Total bilirubin (mmol/l)	10.8 ± 4.2	11.0 ± 4.0	0.823	11.0 ± 4.0	10.7 ± 3.8	0.813	0.788
AST (IU/L)	20.7 ± 6.6	20.8 ± 6.4	0.949	21.3 ± 7.3	21.5 ± 6.9	0.927	0.684
ALT (IU/L)	23.7 ± 7.4	23.4 ± 7.5	0.916	22.9 ± 6.2	23.2 ± 6.1	0.832	0.909
ALP (U/L)	171.4 ± 38.5	171.5 ± 38.6	0.995	178.8 ± 31.8	179.0 ± 31.9	0.974	0.422
BUN (mg/dL)	11.1 ± 3.3	11.4 ± 3.3	0.749	12.1 ± 2.9	12.0 ± 3.0	0.894	0.220
Creatinine (mg/dL)	0.7 ± 0.1	0.7 ± 0.1	0.915	0.8 ± 0.1	0.8 ± 0.1	0.897	0.451
Total cholesterol (mg/dL)	141.3 ± 17.3	141.1 ± 17.4	0.970	137.3 ± 17.4	137.3 ± 17.2	0.994	0.403

* Paired samples *t*-test between baseline and day-56 (Within group); ** Two sample t-test for between group comparisons of post-treatment (day 56) values. ARE, ashwagandha root extract; ALT, alanine aminotransferase; ALP, alkaline phosphatase; ANC, absolute neutrophil count; AST, aspartate aminotransferase; BUN, blood urea nitrogen; PCV, packed cell volume; RBC, red blood cell; TLC, total leukocyte count; SD, standard deviation

## Discussion

### Primary outcome

ARE supplementation for 56 days resulted in significant improvements in core ADHD symptoms, with consistent reductions across inattention, hyperactivity–impulsivity, and total ADHD-RS-IV scores compared with placebo. The lack of baseline differences supports that these effects are attributable to the intervention, indicating a broad impact across symptom domains.

From a mechanistic perspective, these findings are consistent with emerging evidence linking ADHD symptom expression to dysregulation of stress-responsive neurobiological systems [33]. Children with ADHD have been shown to exhibit HPA axis activity, impaired stress regulation, and heightened emotional reactivity, all of which are associated with attentional control and behavioral inhibition [34]. Ashwagandha root extract has been widely studied for its adaptogenic properties, including its capacity to modulate cortisol secretion and influence neurotransmitter systems involved in attention and executive functioning [12,35]. While mechanistic mechanisms were not directly assessed, the reductions in ADHD-RS-IV scores are consistent with the possibility that improved stress regulation contributes to behavioral benefits in pediatric ADHD.

The present findings add to the limited clinical evidence evaluating herbal interventions for ADHD. Much of the existing pediatric research has examined multi-ingredient formulations or focused on general cognitive outcomes rather than on validated ADHD symptom scales, thereby limiting clinical interpretation [21]. This study adds to the limited pediatric evidence on herbal interventions for ADHD by evaluating a standardized, single-agent extract using a validated, DSM-aligned outcome measure. Pharmacological treatments remain first-line therapy for moderate to severe ADHD; however, concerns related to tolerability, long-term use, and persistent symptoms have increased interest in complementary approaches [36]. Within this context, the significant reductions in ADHD-RS-IV scores observed in the present study support further investigation of ARE as a potential adjunctive option for children and adolescents.

### Secondary outcomes

ARE supplementation was associated with significant improvements in executive functioning across behavioral, emotional, and cognitive domains, as reflected by BRIEF-2 scores, suggesting a broader impact on higher-order regulatory processes relevant to daily functioning in ADHD. The concurrent improvements in both symptom severity and executive regulation observed in the present study are therefore notable, as previous research has highlighted dissociations between reductions in core ADHD symptoms and changes in executive functioning [37]. Executive processes assessed by the BRIEF-2 are closely related to prefrontal cortical networks that are sensitive to stress and emotional dysregulation [38]. The associated improvements in ARE and BRIEF-2 outcomes are consistent with a potential effect on executive functioning.

In addition, improvements in sleep disturbance and anxiety scores indicate beneficial effects beyond core symptoms, addressing commonly associated comorbid features that can exacerbate ADHD severity [39]. The observed changes in PROMIS outcomes therefore suggest that improvements in ADHD symptoms and executive functioning occurred alongside broader improvements in emotional wellbeing and sleep-related functioning. These secondary outcomes support the view that ARE may exert a range of effects relevant to the complex clinical presentation of ADHD, warranting further investigation in larger and longer-term trials.

### Safety and efficacy of ashwagandha root extract

The absence of clinically relevant hematological or biochemical changes after 56 days of ARE supplementation, along with stable laboratory markers (liver, renal, hematological, and lipid status), indicated good short-term tolerability without systemic toxicity.

Safety considerations are particularly salient in pediatric ADHD research due to concerns regarding long-term pharmacotherapy and its potential effects on growth, cardiovascular parameters, and neurodevelopment [40]. Reports of hepatic adverse events with ashwagandha have largely involved non-standardized or leaf-based extracts with unclear phytochemical composition [41,42]. The present study used a standardized root-only extract with negligible levels of *withaferin A* and *withanone*, compounds implicated in hepatotoxicity under certain conditions [43]. These findings are consistent with adult trials demonstrating good tolerability, including a 12-month study reporting sustained safety without serious adverse events [44,45]. While extrapolation to pediatric populations requires caution, this concordance strengthens confidence in the short-term safety of ARE under clinical supervision.

## Conclusion

This randomized, double-blind, placebo-controlled study demonstrated that supplementation with ashwagandha root extract over 56 days was associated with significant improvements in core ADHD symptoms, executive functioning, sleep disturbance, and anxiety in children and adolescents. These benefits were observed alongside a favorable safety profile, with no clinically relevant changes in hematological or biochemical parameters. The findings support the potential role of ARE as a well-tolerated complementary intervention in pediatric ADHD and provide a rationale for larger, longer-term clinical trials to further establish efficacy, safety, and optimal use within integrative treatment frameworks.

## Data Availability

The data that support the findings of this study are available from the corresponding author upon reasonable request.

## Personal thanks

The authors thank Ixoreal BioMed Inc., Los Angeles, California, USA, for supplying the KSM-66 ashwagandha root extract used in the study.
